# Board overconfidence and M&A performance: evidence from the UK

**DOI:** 10.1007/s11156-023-01133-8

**Published:** 2023-01-30

**Authors:** Sanjukta Brahma, Agyenim Boateng, Sardar Ahmad

**Affiliations:** 1 Glasgow School for Business and Society, Glasgow Caledonian University, Glasgow, G4 0BA UK; 2 Leicester Castle Business School, De Montfort University, Leicester, UK; 3 University of Liverpool Management School, University of Liverpool, Liverpool, UK

**Keywords:** Mergers and acquisitions, Overconfidence, Board gender, Acquisitiveness, G34, G41

## Abstract

This paper investigates the relationship between board overconfidence and mergers and acquisitions (M&A) performance based on 754 M&A deals in the UK from 2002 to 2018. Employing three proxies to measure overconfidence, namely, fraction of male directors on the board, multiple acquisitions and merger characteristics, our results suggest that a higher fraction of male directors on the board and multiple acquisitions lead to poor M&A performance. The results also show that multiple acquirers’ deals generate higher returns than subsequent deals and this is due to self-attribution bias. In terms of merger characteristics, this study has found that, when overconfident acquirers use cash as the method of payment or when they embark on diversifying M&A, it leads to poor M&A performance. The results are robust across both univariate and multivariate analyses and also across alternate measures of post-merger performance. The findings of this study have important policy implication with regard to the ratio of male directors, number of acquisitions and the method of payment.

## Introduction

Mergers and acquisitions^1^ (M&A) are amongst the most common corporate investment decisions made by managers (Harford and Li 2007; Guo et al. 2018). Despite its popularity stylised facts indicate that target firms’ shareholders gain, while the acquirer firms’ shareholders experience significant losses in the short run.^2^ Evidence also suggests that even the long-run shareholder returns are mostly negative.^3^ For instance, earlier studies by Agrawal et al. (1992) show that acquirer shareholders suffer substantial losses of 10% in the five-year post-merger period. Loughran and Vijh (1997) report mixed evidence on the long run post-merger performance. They find that stock mergers suffer significant negative returns of 25% whereas cash tender offer mergers make significant positive returns over the 5-year post-merger period. However, Mitchell and Stafford (2000) on the other hand did not find any significant long-term abnormal performance.^4^ Most of the past empirical efforts examining how corporate boards affect M&A outcomes have predominantly employed agency problems amongst managers and shareholders (see, Jensen and Meckling 1976; Seth et al. 2000) and have typically maintained that economic factors rather than behavioural factors drive M&A outcomes (see, Huang et al. 2016).

Notwithstanding this, the importance of the behavioural approach, which embraces the concept of overconfidence offers an important lens to explain post M&A performance. However, research evidence adopting this behavioural approach appears scant, with the exception of notable studies by Doukas and Petmezas (2007), Billet and Qian (2008), Malmendier and Tate (2008) and Guo et al. (2020). Results from these studies are so far mixed and, clearly, further research is required in this area to understand the role of behavioural factors in post-M&A performance. In this paper, we ask whether managerial overconfidence can explain M&A performance using a new proxy for managerial overconfidence, thereby extending prior literature.

Focusing on overconfidence is not new and has been examined before in the context of M&A. For instance, Doukas and Petmezas (2007) use managerial acquisitiveness and insider dealings to measure overconfidence while Malmendier and Tate (2008) use longholder (late exercise of executive stock options) CEO as a trait for overconfidence. Tang et al. (2020) report that CEO overconfidence is positively linked to post merger firm value and this is more pronounced for young CEOs whereas female CEOs are risk-averse and show lower firm volatility and leverage. Similarly, Guo et al. (2020) have taken manager’s relative salary as the measure of overconfidence. Contributing to this stream of research, our study focuses on managerial overconfidence instead of just CEO overconfidence commonly used in the literature (e.g.Malmendier and Tate 2008; Doukas and Petmezas 2007; Chen et al. 2022; Hsu et al. 2022; Guan et al. 2022; Ismail and Mavis 2022; Malhotra et al. 2022). It is argued that, focusing on managerial overconfidence instead of CEO overconfidence provides a better proxy as M&A deals would be the board of directors’ collective decision. For instance, in a recent study, Li and Tong (2022) argue that managerial overconfidence is associated with the type of CEO being recruited. Furthermore, in many large firms the characteristics of board members exert significant power in decision-making and could also affect the post-merger performance.

We use three different measures to establish the level of managerial overconfidence. The first measure of overconfidence, fraction of male directors, is driven by both psychological and behavioural finance models which have established that, in the fields of finance and investment, men are more likely to be overconfident and trade more excessively compared to women (see Barber and Odean 2001; Croson and Gneezy 2009). This prediction has been tested in the context of investors by analysing their trading behaviour based on gender. Barber and Odean (2001) show that men are more overconfident investors than their female counterparts. Such overconfident investors misjudge the accuracy of information they receive and possibly may trade even when the forecasted gains are negative (Odean 1998; Barber and Odean 2001). Moreover, research evidence indicates that overconfident investors are more likely to hold riskier investment portfolios in comparison to rational investors, but with a similar level of risk aversion (Croson and Gneezy 2009). Furthermore, in the context of acquisition and other risky investments, empirical evidence shows that women are risk averse as compared with men (Farrell and Hersch 2005; Levi et al. 2014). The risk averse behaviour of female directors has been linked with lower firm risk (Farrell and Hersch 2005) and less aggressive acquisition strategies (Levi et al. 2014). As M&A involve extensive discussion at board level and approval by individual directors, the proportion of board gender representation could play a role and is an important factor to be considered. Hence, we employ the fraction of male directors on a corporate board as a proxy for overconfidence. This measure of overconfidence is consistent with psychological and behavioural finance models (Barber and Odean 2001; Croson and Gneezy 2009).

For our second measure of overconfidence, we follow Doukas and Petmesaz (2007) and Billet and Qian (2008), who employed managers’ acquisitiveness as a proxy for overconfidence. The logic behind this overconfidence measure is ascribed to the fact that overconfidence drives managers to undertake multiple acquisitions in a short period of time, where they do not give themselves sufficient time to evaluate the synergies from mergers. Consistent with Odean’s (1998) powerful explanation that greater overconfidence leads to more trading, prior studies contend that multiple acquisitions are indicative of overconfidence, and Malmendier and Tate (2004) maintain that making multiple acquisitions in a year is an indicator of overconfidence.^5^ We classify multiple acquirers to be firms that have completed five or more M&A within 3 years of the first acquisition.

Our third measure of overconfidence is merger characteristics. We use two merger characteristics, diversifying acquisitions and method of payment. Brown and Sarma (2007) and Ferris et al. (2013) examine CEO overconfidence on M&A and find that overconfident CEOs engage in diversifying acquisitions. Drawing on Jensen’s free cash flow hypothesis, Malmendier and Tate (2004) posit that managers with abundant internal resources use cash to finance M&A and sometimes this leads to overpayment. In a similar vein, Doukas and Petmezas (2007) argue that overconfident CEOs with cash reserves and low leverage would undertake value-destroying M&A compared to rationale CEOs. Malmendier and Tate (2008) also advance the argument that overconfident CEOs misjudge the returns produced by their companies and consider that outside investors undervalue their businesses and hence they tend to use cash to finance M&A. Ferris et al. (2013) results concur with this view as they report that overconfident CEOs use cash as the method of payment and engage in multiple M&A.

The choice of the UK is motivated by the fact that it has been the top acquiring nation in Europe over the past two decades with the total value of acquisitions reaching £181.3 billion and £231 billion in 2000 and 2016, respectively.^6^ Despite the COVID 19 pandemic, a survey by Ernst and Young (EY) shows that in 2020 alone M&A deals valued at £347 billion took place in the UK, which was 13% of the global total deals.^7^ Unlike US M&A, UK M&A are distinct as they are characterised by private target firms. Our data shows that 99% of UK targets are private firms. Thus, the dominance of private firms makes the examination of M&A in the UK setting interesting because private targets’ limit the breadth of the acquirer’s search due to lack of information, thereby increasing the risk of acquisition becoming a financial failure. At the same time, Capron and Shen (2007) contend that less information on private targets create more value-creating opportunities by exploiting private information. In short, since private firms are not under scrutiny by shareholders and institutional investors, they are not as transparent in their activities as their public counterparts. Consequently, their valuation would be largely grounded on acquirer managers’ views about potential synergies and cash flows in the future. This provides a model testing ground to gauge whether M&A are driven by managerial overconfidence.

This paper makes two significant contributions to the literature. First, we use a novel measure, i.e., the relative fraction of male directors, as a measure for overconfidence to explain how male versus female behavioural traits affect the economic outcomes of M&A. The results suggest that a higher fraction of male directors generates poor announcement returns in the short term and negative post-merger returns in the long run. To the best of our knowledge, this study is the first to examine the connection between overconfidence proxied as a fraction of male directors on the board and M&A performance. The present research adds to the growing number of studies on how behavioural differences between genders matter in corporate investment decisions. Second, the performance of M&A has been an overarching research theme over the past few years, yet most M&A have failed financially (see, Ravenscraft and Scherer 1987), and we do not fully understand the reasons for their failure. This study sheds light on the factors that may influence M&A performance. We show that managerial overconfidence, measured by using three different proxies of overconfidence, affects the economic outcomes of M&A investments and that the fraction of males in the boardroom and frequency of acquisitions and merger characteristics are sources of managerial over-optimism in their investment decisions. Consequently, issues such as board gender diversity in the UK boardrooms being advocated by civil societies, and the reports by Lord Davies in 2011 and Hampton Alexander in 2017, should be seriously considered by practising managers and policy makers.

The remainder of the paper is structured in the following way. In Sect. 2, we present the theoretical and empirical literature. In Sect. 3 we present our testable hypotheses, Sect. 4 outlines our data and methodology. Section 5 presents our main results and analyses. We present our conclusion in Sect. 6.

## Literature review

### Gender differences and overconfidence

The association between corporate investment decisions and gender differences is a subject of immense interest for researchers from both psychology and finance (Beyer 1990; Jiankoplos and Bernasek 1998; Barber and Odean 2001). Both psychological and behavioural finance literatures have recognised that men are more overconfident than women. An early study by Beyer (1990) shows that self-attribution bias is higher in men. Similarly, using the amount of trading activity engaged in by both men and women to measure overconfidence, Barber and Odean (2001) find that men trade 45% more than women, and this remains the case even when the expected net returns from trade are negative. They therefore conclude that men are more overconfident regarding their abilities to trade than women are. Hence, having greater overconfidence leads to greater trading from a psychological standpoint. Moreover, Lenney (1977) and Malmendier and Tate (2005, 2008) argue that women are likely to see future results in a more conservative sense than men and hence they demonstrate a reluctance to accept difficult and risky strategies.

Grounded on the above arguments, several studies have looked into the link between male and female overconfidence (i.e., gender differences) and risk-taking behaviour (Jiankoplos and Bernasek 1998). The results indicate that women are risk averse in financing decisions as they are less likely to be overconfident compared to men (Niederle and Vesterlund 2007; Barber and Odean 2001). A number of studies, such as Farrell and Hersch (2005), Dowling and Aribi (2013) and Levi et al. (2014), have provided support for the point of view that women investors are risk averse. For example, Farrell and Hersch (2005) find firms’ risk is negatively associated with the number of female directors on corporate boards. Recent studies by Levi et al. (2014) report that female directors are likely to undertake less hostile acquisition strategies and that they often pay lower bid premiums. In contrast to this, Huang and Kisgen (2013) document that male directors undertake more mergers with lower returns and issue more debt compared to their female counterparts. In this context, Lee et al. (2021) report that excessive debt-based CEO pay may lower firms’ investment on value enhancing policies like R&D investment and hence might alleviate long-term firms’ achievements.

Few studies have looked into board gender diversity and M&A. For example, Ben-Amar et al. (2013) find that demographic and strategic diversity can have mixed effects, depending on other contextual factors such as firms’ ownership concentration. Parola et al. (2015) show that top management team (TMT) diversity has a favourable impact on pre-integration performance in comparison to post-integration performance. He et al. (2020) report that acquirer firms’ past association with targets is positively related to long-term profitability and this is more explicit when target specific knowledge and skill are critical for merger success. In a related study, Ravaonorohanta (2020) documents that a gender-diverse board has a favourable impact on merger performance and reduced acquisition premiums in firms when there are fewer female members in the executive team, and speculates that managerial overconfidence may be a potential explanation. Our study is different from the above studies as none of these studies on gender and M&A have considered higher male representation on the board as a source of overconfidence. Given the theoretical and empirical evidence discussed above, this is a reasonable supposition.

### Managerial overconfidence and M&A

Psychology literature advocates that people are overconfident when they think that the situation is under their command and something to which they are extremely dedicated (Langer 1975; March and Shapira 1987; Weinstein and Klein 2002). Several psychology and behavioural finance studies have identified this source of overconfidence as self-attribution bias, which refers to the trend where managers link successful firm strategies to their own achievements but failures to external factors (Kahneman and Tversky 2000). Self-attribution bias has been observed in different areas of finance. For example, Daniel et al. (1998) attribute investor overconfidence to this bias; Gervais and Odean (2001) state that self-attribution bias instigates traders to develop overconfidence; while Hilary and Menzly (2006) document that those analysts with self-attribution bias become overconfident through their short-term success. Other studies on managerial overconfidence show that overconfident managers delay loss reporting by using less conservative accounting and this factor could not be mitigated by external corporate governance (Ahmed and Duellman 2013), give out higher audit fees for firms that do have a strong audit committee and tend to hire non-industry specialist auditors (Duellman et al. 2015), overconfident managers with higher managerial skills pay significantly lower audit fees and could pay significantly higher audit fees where there is board oversight (Mitra et al. 2019).

Regarding the relationship between overconfidence and M&A, Roll’s (1986) study was the first attempt to assess the impact of managerial overconfidence and to document that takeover fights could cause winner’s curse. Roll (1986) put forward the hubris hypothesis, which advocates that acquirer firms’ managers could be overconfident and overestimate the potential gains of a takeover, thereby destroying firm value. After Roll’s (1986) seminal paper was published, several researchers investigated the impact of managerial hubris on M&A. For instance, Hayward and Hambrick (1997) examine Roll’s hubris hypothesis and find evidence to suggest that CEO overconfidence leads to overbidding of target firms. Malmendier and Tate (2005) investigate the link between M&A and managerial overconfidence in the context of US data, and find greater mean acquisitiveness by overconfident CEOs. In the context of the UK, Doukas and Petmezas (2007) look into managerial overconfidence of private targets and find that overconfident acquirers obtain lower acquisition announcement returns in comparison to rational acquirers and demonstrate weak long-term performance. Conversely, using data from Chinese listed companies, Guo et al. (2020) find that market reaction is favourable for M&A deals in firms with overconfident managers. In similar vein, Pan et al (2019) show that managerial overconfidence leads to positive M&A premium and this result is more prominent for firms with higher debt capacity.

The above studies posit that CEOs suffer from cognitive biases and hence they feel that they have excellent decision-making skills, which are reflected in their underestimation of merger risks and overestimation of synergistic gains from acquisitions. Driven by self-attribution bias, CEOs become overconfident through the success of their first acquisitions and undertake multiple acquisitions within a short time span. Malmendier and Tate (2004) document that this overestimation stems from managers’ belief that they have superior leadership attributes. Doukas and Petmezas (2007)^8^ and Billet and Qian (2008) show that multiple acquisition is a sign of managerial overconfidence and this is manifested by the fact that deals with five or more acquisitions in a span of three years are more strongly linked with poor value creation than first deals. Multiple acquisitions by corporate managers are equivalent to overconfident analysts who believe in their superior stock-picking skills that actually lead to subsequent poorer returns (Doukas and Petmezas 2007). Multiple acquirers are analogous to models of investor overconfidence that forecast high trading volume by overconfident traders (Odean 1998). The link between multiple acquisitions and overconfidence can also be traced to the notion that overconfidence increases the likelihood of succeeding in contests. More recently, Li et al. (2021) analyse the difference in the time between M&A deals in 81 countries and document that multiple mergers undertaken in a short span of time generate lower abnormal returns. However, none of these extant studies have examined the effect of managers’ overconfidence on M&A in the post-financial crisis phase in the UK, which was characterised by significant regulatory overhaul, and this study addresses that gap.

### Empirical evidence on merger characteristics

M&As can be distinguished by the method of payment, i.e. cash versus stock payment, and the nature of the M&A, i.e. diversifying versus related M&As. Jory et al. (2020) report that stock acquirers that are overvalued, earn lower profit and face financial limitation are more prone to stock price crash. Extant studies manifest that M&A characterised by cash payment are driven by managerial overconfidence. These studies contend that managers pursue acquisitions when they have ample internal resources and hence use cash to finance their mergers instead of equity (Brown and Sarma 2007; Malmendier and Tate 2008; Ferris et al. 2013). In addition, Doukas and Petmezas (2007) and Malmendier and Tate (2008) also contend that managers driven by overconfidence based on their leadership attributes and chosen investment projects may overestimate the value of their firms and believe that their equity is undervalued. In a similar vein, Malmendier and Tate (2004) suggest that overconfident CEOs will undertake value-destroying acquisitions if the forecasted gains and equity undervaluation are significantly large. These types of M&A tend to use cash as the payment method. Given these discourses, we use cash financed M&A as our third measure of managerial overconfidence.

Ferris et al. (2013) argue that diversifying mergers are risky as the firm has no experience in that sector, so managers depend more on their instincts, which may be linked with greater behavioural biases (Kahneman 2003), and hence managerial overconfidence can push this type of merger. Morck et al. (1990) document negative announcement period returns for diversifying M&A. Following these studies, we relate diversifying M&A to managerial overconfidence.

## Hypotheses development

### Overconfidence and gender

The empirical evidence discussed above indicates that males are more overconfident than females (e.g. Barber and Oden 2001). With overconfidence linked to a propensity for taking excessive risk, it could be hypothesised that a higher fraction of male directors on the board could lead to increased managerial overconfidence. We argue that this increased managerial overconfidence in unbalanced gender-diverse boards manifests in lower announcement period returns of the acquirer firms and also leads to poor post-merger performance. Apart from the above theoretical and empirical evidence on gender diversity, this supposition is also based on further empirical findings that a greater number of female directors is linked to improved earnings quality (Gul et al. 2011), better supervision of firm activities (Adams and Ferreira 2009), more careful policy choice (Milliken and Martins 1996) and better firm performance (Brahma et al. 2021). Based on the above arguments, we contend that a higher fraction of male representation in the boardroom is likely to cause poor merger outcomes and hence we hypothesise the following:

#### H_1a_

Boards with a greater fraction of male directors will lead to lower returns for the acquirer firms following the M&A announcement.

#### H_1b_

Boards with a greater fraction of male directors will cause negative returns in the long run.

### Overconfidence and self-attribution bias

As documented in Sect. 2, extant studies have associated multiple acquisitions as a sign of managerial overconfidence (Doukas and Petmezas 2007; Malmendier and Tate 2004; Billet and Qian 2008). After a successful first acquisition, overconfident managers engage in subsequent multiple acquisitions, driven by self-attribution bias; however, they have less time to evaluate the potential synergies from subsequent acquisitions. Hence, we hypothesize that more multiple acquisitions, following the first acquisition, in a short period can destroy the firm value of acquirers.

#### H_2a_

Announcement period acquirer returns are higher for single acquirers in comparison to multiple acquirers and also higher for multiple acquirers’ 1st deal compared to their 5th or more deals.

#### H_2b_

Post-merger combined firm returns are higher for single acquirers in comparison to multiple acquirers and also higher for multiple acquirers’ 1st deal compared to their 5th or more deals.

### Overconfidence and merger characteristics

As discussed in Sect. 2, overconfident managers with abundant internal resources would use cash as the method of payment. Overconfident managers also perceive that their equity is undervalued and hence finance their acquisitions with cash. In addition, overconfident CEOs engage in more diversifying acquisitions than related acquisitions. M&A literature suggests that managers use stock as the method of payment when equity is overvalued and use cash when it is undervalued (Asquith et al. 1983). However, Doukas and Petmezas (2007) find that overconfident managers^9^ use cash even when the equity is overvalued. This is in line with Jensen’s (1976) free cash flow hypothesis, that is, firms with abundant internal resources tend to engage in acquisitions and use cash as the payment method.

Driven by cognitive biases and illusion of control (Malmendier and Tate 2004), overconfident managers believe that they have superior ability to deal with complicated activities like diversifying acquisitions, that is, acquisition of firms in a different line of business. When managers engage in diversifying deals they have to depend more on their own judgement, which may lead to inaccurate estimation of the potential synergies that are likely to be generated from M&A. This is in line with Kahneman’s (2003) study, which suggests that managers rely on their own intuitive judgement when there is sparse information like in this case of diversifying acquisitions.

Given this theoretical and empirical evidence, we develop the following hypotheses based on M&A deal characteristics which are method of payments and related versus diversifying acquisitions.

#### H_3a_

Cash-financed M&A generate lower announcement period returns than stock-financed M&A.

#### H_3b_

Cash-financed M&A generate lower long run post-merger returns than stock-financed M&A.

#### H_4a_

Diversifying M&A generate lower announcement period returns than related M&A.

#### H_4b_

Diversifying M&A generate lower long run post-merger returns than related M&A.

## Data and methodology

### Sample and data sources

The data for M&A and merger characteristics has been taken from the Securities Data Corporation (SDC) database. All other data on share price and firm characteristics have been collected from Datastream. We have applied the following criteria for sample selection: (i) The acquirer firms belong to publicly traded firms in the UK; (ii) Target firms belong to UK private firms (including subsidiaries) and public firms. Private targets are taken as they are difficult to value and hence provide an ideal testing ground for managerial acquisitiveness. This is because private targets are based on managers’ own evaluation of possible synergies from the M&A, whereas public target firms with larger investor base, are easier to value as they are more transparent due to constant scrutiny by market analysts. Within the sample period given below, we find that 99% of M&A deals are comprised of deals where the target firms are private companies. This is in line with the studies by Doukas and Petmezas (2007) and Dowling and Aribi (2013). (iii) The acquirer must obtain more than 50% of the target firms’ shares after the completion of the takeover deal; (iv) We have also excluded financial and utility firms from the sample as these firms are subject to economic regulations and hence managerial overconfidence may not be a significant factor for their takeover decisions. Based on the above criteria, we have obtained 754 M&A deals from 2002 to 2018; (v) The first measure of overconfidence is obtained by calculating the fraction of male directors on the board; and (vi) In order to evaluate our other measure of overconfidence, we follow Doukas and Petmezas (2007) and define multiple acquirers to be firms that complete five or more acquisitions within a span of three years.

Table 1, Panel A, presents the M&A sample among private and public targets segregated by the acquisitiveness of the acquirer, related versus diversifying acquisitions, cash, stock or mixed payment and transaction value; and Panel B reports the descriptive statistics of other board variables.

**Table 1:** Descriptive statistics

Type of acquisition	Number of acquisitions	Mean transaction value (£ million)	Median transaction value (£ million)	Cash	Stock	Mixed	Related	Diversifying	Deal size/bidder’s market capitalization
*Panel A*									
	N			N	N	N	N	N	N
All deals	754	158.1	38.5	316	136	293	250	504	0.0624
Private	744	156.8	38.3	311	136	289	247	497	0.0625
Public	10	253.4	41.7	5	0	4	3	7	0.0503
Single	331	226.5	59.4	116	70	145	133	198	0.0640
Multiple	423	111.8	31.7	200	66	148	117	306	0.0468
Deal value > 20 million	497	234.89	79.8	213	90	186	153	344	0.0883

	Number of acquisitions	Mean	Median	Standard deviation	Max	Min
*Panel B*						
Fraction of male directors	754	0.78	0.72	0.05	1	0.62
CEO duality	754	0.24	0	0.062	1	0
Board size	754	9.2	9	0.045	15	6
Board independence	754	5.31	5	0.11	24	2
Tobin's Q	754	0.65	0.68	0.07	0.87	0.21
Leverage	754	22.1	35.3	0.3	83.7	0

Panel A of the table shows the distribution of deals in terms of private and public deals and between single and multiple deals. Panel B shows the summary statistics of other independent and control variables

The results suggest an interesting trend, which is that a large fraction of UK acquirers (about 56%, 423 deals) engage in multiple acquisitions and the overwhelming majority (99%, 744 deals) are linked to private deals. In addition, a staggering 42% (316 deals) of all M&A are completed in cash and 18% (136 deals) in stock; the rest are financed by mixed financing. Multiple acquirers make greater use of cash (47%, 200 deals) while only 16% (66 deals) of the multiple acquirers use stock. Consistent with Doukas and Petmezas (2007), this suggests that equity overvaluation does not drive UK mergers. The higher percentage of cash transactions by multiple acquirers supports the notion that overconfident managers show an inclination towards internal financing as they recognise their companies as being undervalued.^10^ This result also implies that managerial overconfidence could impact on firm decision-making (Brown and Sarma 2007; Malmendier and Tate 2008; Ferris et al. 2013). The result also shows that 67% (504 deals) of all acquisitions are diversifying acquisitions, while only 33% (250 deals) are related acquisitions. Within the multiple M&A deals, the majority, i.e. 72% (306 deals), are diversifying M&A. This result validates the findings of empirical studies that overconfident managers with abundant internal resources engage in diversifying acquisitions rather than related acquisitions (Morck et al. 1990; Ferris et al. 2013). We further screen the sample by taking M&A deals where the transaction size is £20 million or more. This is shown in the last row of Panel A. As the Table shows, 497 out of 754 M&A deals comprises of transaction size of £20 million or more and 488 of these deals are private target firms and the rest of the deals constituting about nine are public target firms. The median transaction value of these large deals is £79.8 million. Hence, it can be concluded that the majority of the private target deals, that is, 65% (488 out of 744) are large deals. We have also analysed the size of the acquisition relative to bidder’s market capitalisation. This is shown in the last column of Panel A in Table 1. The results show that for the full sample the average size of the acquisition relative to the bidder’s market capitalisation is 0.0624, which is about 6% of the bidder’s market capitalisation. For large deals at or above £20 million this figure is 0.0883 implying that on average for large deals the average size of the acquisition to bidder’s market capitalisation is about 8.8%.

Panel B of Table 1 shows that the mean fraction of male directors in the board is 0.78, implying that on average 78% of board members are male directors. Over the sample period, boards in the UK have become more gender diverse as the fraction of male directors has fallen and the number of female directors has increased. Similar UK board gender diversity trend has also been observed by Dowling and Aribi (2013) in the context of UK M&A and Brahma, Nwafor, Boateng (2021) in the context of FTSE100 firms.

Table 2 shows the spread of the M&A deals around the time period selected. As evident from the table, in most years cash deals and diversifying acquisitions were higher than related acquisitions. The results show that percentages of cash payments are higher in most years for multiple acquirers than for single acquirers. In addition, the percentage of related deals is higher for single acquirers (40%, 133 out of 331 deals) than for multiple acquirers (28%, 117 out of 423). This result has substantiated the results of Table 1 that multiple acquirers display managerial overconfidence and conduct M&A with cash rather than stock, and also engage in M&A with firms that are in unrelated sectors.

**Table 2:** M&A deals by year

Year of completion	No. of acquirers		Single (S)										Multiple (M)									
	S	M	C		Sk		Mx		R		D		C		Sk		Mx		R		D	
			N	%	N	%	N	%	N	%	N	%	N	%	N	%	N	%	N	%	N	%
2018	10	11	8	9	2	3	0	0	2	2	6	5	5	2	3	4	3	3	5	4	6	2
2017	14	10	9	10	2	3	3	2	4	4	10	8	6	3	0	0	4	4	3	2	7	2
2016	16	22	6	6	5	8	5	4	9	8	2	2	17	8	2	3	3	3	6	5	16	5
2015	9	49	4	4	3	5	2	2	2	2	7	6	34	16	4	5	11	11	8	6	41	12
2014	19	32	8	9	3	5	8	6	9	8	3	2	15	7	4	5	13	13	9	7	23	7
2013	15	36	5	5	5	8	5	4	8	7	7	6	17	8	4	5	15	15	6	5	30	9
2012	16	41	9	10	2	3	5	4	7	6	4	3	27	13	7	9	7	7	12	9	29	9
2011	18	40	7	8	7	12	4	3	7	6	11	9	19	9	8	11	13	13	11	9	29	9
2010	12	30	4	4	4	7	4	3	4	4	5	4	15	7	5	7	10	10	10	8	20	6
2009	11	22	3	3	0	0	8	6	7	6	4	3	8	4	7	9	7	7	7	5	15	4
2008	26	40	7	8	8	13	11	9	9	8	6	5	7	3	7	9	14	14	11	9	29	9
2007	37	34	10	11	9	15	18	14	21	18	16	13	14	7	6	8	13	13	10	8	24	7
2006	25	27	3	3	4	7	18	14	8	7	7	6	9	4	4	5	15	15	14	11	13	4
2005	21	31	4	4	3	5	12	10	6	5	15	12	9	4	4	5	17	17	8	6	23	7
2004	19	22	4	4	3	5	12	10	11	10	8	7	5	2	6	8	11	11	5	4	17	5
2003	2	11	1	1	0	0	1	1	0	0	2	2	5	2	5	7	1	1	3	2	8	2
2002	10	6	1	1	0	0	9	7	0	0	9	7	3	1	0	0	3	3	1	1	5	1
Totals	280	464	93	100%	60	100%	99	100%	114	100%	101	100%	215	100%	76	100%	160	100%	129	100%	335	100%

This table presents the distribution of M&A deals across different years and across single and multiple acquirers. The table also shows the yearly spread of the deals for the single (S) and multiple (M) acquirers in relation to payment method, cash (C), stock (Sk) and mixed (Mx) and in terms of related (R) versus diversifying (D) acquisitions

### Measurement of short-term announcement period returns

This study has adopted event study methodology to examine short-run announcement period returns following Brown and Warner (1980, 1985). The efficient market hypothesis suggests that securities earn normal returns in the absence of any event announcements. So, abnormal returns following the announcement of an event reveal the impact of that event announcement on the security returns. Abnormal returns are estimated as the difference between actual return and normal (benchmark) return in the absence of the event.

1 $${\text{AR}}_{i,t} = R_{i,t} - E\left( {R_{i,t} } \right)$$

In this study we calculate the benchmark return using the OLS market model following Brown and Warner (1985). Under the OLS market model, we regress the actual returns of the sample firms in the estimation period with regard to the actual returns of a market index. In this case, we have taken FTSE100 returns as the market return.

2 $$R_{i,t} = \alpha_{i} + \beta_{i} R_{m,t} + \varepsilon_{i,t}$$

3 $$E\left( {R_{i,t} } \right) = \hat{\alpha } + \hat{\beta }R_{m,t}$$

The average abnormal returns (AAR) for the 11-day event window across the N portfolios are analysed using the following equation:

4 $$AAR_{t} = \frac{{\mathop \sum \nolimits_{i = 1}^{n} AAR_{i,t} }}{N}$$

The cumulative average abnormal returns (CAAR) indicate the overall implication of the announcement of M&A. In addition, CAAR also indicate the effect of the event over different event windows.

The CAAR across different event windows are calculated as follows.

5 $$CAAR_{t1,t2} = \sum\limits_{t1}^{t2} {AAR_{t} }$$

In this study, we have taken two short event windows, CAAR (− 2, + 2) and CAAR (− 1, + 1), as they are the most popular event windows for M&A research (Andrade et al. 2001).

### Measurement of long-run performance

Following Lyon et al. (1999) and Datta et al. (2013), we have measured the buy-and-hold abnormal return (BHAR) to calculate post-merger long-run stock price performance. We have examined the monthly BHARs as many studies assert that the BHAR is in line with the true investor experience (Barber and Lyon 1997; Lyon et al. 1999; Datta et al. 2013).

#### Buy-and-hold abnormal return

The BHAR of security *i* for the holding period T is calculated as follows:

6 $$BHAR_{i,t} = \mathop \prod \limits_{t = 1}^{T} \left( {1 + R_{i,t} } \right) - \mathop \prod \limits_{t = 1}^{T} \left( {1 + E\left( {R_{i,t} } \right)} \right)$$

In Eq. 6, $R_{i,t}$ is the return of security i at month T and $E(R_{i,t} )$ is the expected or normal monthly return based on a benchmark model. The number of months in the holding period after the completion month of the event is denoted by T. This study has analysed 12-months and 24-months holding period BHARs following the event completion month. The ABHAR for the sample of N firms for a particular holding period T (12 or 24 months post-merger) is calculated as follows:

7 $$ABHAR_{T} = \frac{1}{N}\sum\limits_{i = 1}^{N} {BHAR_{i,T} }$$

##### Size and market-to-book value matched control firm for calculating BHAR

Following Fama and French (1992), we identify a control firm for each of our sample M&A firms by matching them in terms of size (market capitalisation) and market value to book value (MV/BV) ratio. First, 25 size and MV/BV control portfolios are constructed by grouping the constituent firms in the market index into five portfolios on the basis of size (market capitalisation). They are then divided into five portfolios on the basis of their MV/BV ratio. The control portfolio is first selected by matching the portfolio that has the MVs closest to the sample firm. From this portfolio, the control firm is selected that matches the sample firm’s size and MV/BV in the month of M&A completion. This control firm return is taken as the expected benchmark return $E(R_{i,T} )$.

##### Propensity score-matched control firm for calculating BHAR

To determine the robustness of the BHARs obtained from size and market-to-book matched control firms, we have calculated the propensity score-matched control firm following Li and Zhao (2006). This methodology is dependent on determining the control firm on the basis of scalar similarity measure between the sample firm and control firm (Rosenbaum and Rubin 1983). The probability of a firm undergoing an event (M&A) based on a set of explanatory variables is denoted by the propensity score, p(x).

8 $$p\left( x \right) = pr\left( {D = 1|x} \right)$$

In Eq. 8, D = 1 for firms that have undergone M&A and D = 0 for non-M&A firms. The explanatory variables selected to calculate the propensity score are important because the score should indicate both the propensity of the control firm to engage in M&A and post-merger performance (Li and Zhao 2006). The propensity score is calculated using the following equation.

9 $$p\left( x \right) = \beta_{0} + \beta_{1} \;Momentum + \beta_{2} \;Size + \beta_{3} {\raise0.7ex\hbox{$M$} \!\mathord{\left/ {\vphantom {M B}}\right.\kern-0pt} \!\lower0.7ex\hbox{$B$}} + \beta_{4} \;DE + \varepsilon_{i}$$

The model in Eq. 9 is based on Akaike and Bayesian information criteria where the dependent variable is a dummy that takes the value 1 if the event (i.e. M&A) has taken place and 0 otherwise. For each sample firm, the control firm is matched with the nearest propensity score, that is, the closest probability to conduct M&A. The results of propensity matched BHARs are shown in Table 5.

### Determinants of M&A returns

The factors determining the short run CARs and the post-merger BHARs are estimated using the following pooled regression model.

10 $$\begin{aligned} Performance_{i} = &\; \alpha_{i} + \beta_{1} \;Fraction\;of\;male\;directors_{i} + \beta_{2} \;Multiple_{i} + \beta_{3} \;Cash_{i} \\ & + \beta_{4} \;Stock_{i} + \beta_{5} \;Related_{i} + \beta_{6} \;Controls_{i} + \varepsilon_{i} \\ \end{aligned}$$

11 $$\begin{aligned} Performance_{i} = &\; \alpha_{i} + \beta_{1} \;Fraction\;of\;male\;directors_{i} + \beta_{2} \;Multiple_{i} \\ & + \beta_{3} \;Fraction\;of\;male\;directors*Multiple_{i} + \beta_{4} \;Cash_{i} + \beta_{5} \;Stock_{i} + \beta_{6} \;Related_{i} \\ & + \beta_{7} \;Controls_{i} + \varepsilon_{i} \\ \end{aligned}$$

$Performance_{i}$ in Eqs. 10 and 11 refers to short-run CAARs across different event windows for the *i*th firm in the M&A sample and 12-months and 24-months post-mergers $BHAR_{i}$ of firm *i* in the M&A sample. The dependent, independent and control variables in Eqs. 10 and 11 are defined in Table 3.

**Table 3:** Variable definitions

Variable name	Definition
*Dependent variable*	
CAR (− 1, + 1)	Cumulative Abnormal Returns of the acquiring firms in the 3 days window of the announcement of M&A
CAR (− 2, + 2)	Cumulative Abnormal Returns of the acquiring firms in the 5 days window of the announcement of M&A
BHAR 12	12 months Buy and Hold Abnormal Returns following the date of merger completion
BHAR 24	24 months Buy and Hold Abnormal Returns following the date of merger completion
*Independent variables*	
Fraction of male directors	Number of male directors in the board divided by board size
Multiple	Dummy variable that takes the of value of 1 if the firm has completed five or more acquisitions within three years of the first acquisition
Cash	Dummy variable that takes the value of 1 if cash is used as the method of payment and 0 otherwise
Stock	Dummy variable that takes the value of 1 if stock is used as the method of payment and 0 otherwise
Related	Dummy variables that takes the value of 1if the merger has taken place within the same industry and 0 otherwise
*Control variables*	
CEO duality	Dummy variable that takes the value of 1 if the CEO is also the Chairman of the firm and 0 otherwise
Board size	Number of total directors on the board
Board independence	Number of independent directors
TobinsQ	A market-based measure which is measured as the book value of total assets minus the book value of common equity plus the market value of common equity divided by the book value of total assets
Leverage	A ratio measuring total debt to total assets

Table 4 presents the correlation matrix. All the correlation coefficients show an absolute value lower than 0.7. In addition, tests for multicollinearity report that the VIF is below 2, indicating that multicollinearity is not an issue.

**Table 4:** Correlation matrix

	1	2	3	4	5	6	7	8	9	10	11	12	13	14
CAR (− 1, + 1)	1													
CAR (− 2, + 2)	0.4	1												
BHAR 12	0.015	0.025	1											
BHAR 24	0.032	0.027	0.043	1										
Fraction of male directors	0.021	0.018	0.031	0.019	1									
Multiple	− 0.27	− 0.24*	0.03	− 0.21*	− 0.076	1								
Cash	− 0.04	− 0.16	− 0.18*	− 0.2*	0.013	0.134	1							
Stock	0.026	0.031	0.016	0.018	0.051	− 0.68**	0.21	1						
Related	0.016	0.018	0.002	0.011	− 0.11*	0.143	0.05	0.067	1					
CEO duality	0.24***	0.28**	− 0.16*	− 0.15*	− 0.021	0.015	0.024	0.018	0.27	1				
Board size	0.021	0.026*	0.007	0.034*	0.16	− 0.12	− 0.026	0.066**	− 0.051	0.06**	1			
Board independence	0.23*	0.18*	0.25*	0.09	0.44	0.02	− 0.19	0.12	0.34	− 0.17*	0.42*	1		
Tobin's Q	0.19	0.21	0.16	0.14	0.07**	0.145	0.14	0.02	0.17	− 0.031**	− 0.22**	0.27	1	
Leverage	− 0.03	− 0.025	− 0.012	− 0.026	0.18	0.017	0.02	− 0.19	− 0.16	0.016	0.011	− 0.024	0.08**	1

This table presents the correlation matrix of all the variables. ***p < 0.01, **p < 0.05, *p < 0.1

## Results and discussions

Section 5.1 outlines the results of univariate analyses to test our three measures of overconfidence by dividing the sample between above and below the mean value of the fraction of male directors, by dividing the sample between single and multiple acquirers, by dividing the sample between diversifying and related deals, and also by cash and stock deals. Section 5.2 presents the results of multiple regression analyses where the dependent variables are short-run CAARs and long-run BHARs.

### Univariate analyses

#### Merger performance and overconfidence measured by fraction of male directors

Table 5 presents the short-term average announcement period CAARs denoted as CAARs for the event window (− 1, + 1) and (− 2, + 2) and long-run performance average BHARs denoted by ABHARs for 12-months and 24-months, respectively. Panel A shows the CAARs for the entire sample. As shown in Panel A, the short-term announcement period CAARs of the acquirer firms are positive and significant at 1% levels for both the event windows (− 1, + 1) and (− 2, + 2). The positive CAARs support the findings of other literatures on M&A that have reported positive short-term gains from M&A (Antoniou et al. 2007; Datta et al. 2013).

**Table 5:** Univariate analysis

	CAAR (− 1, + 1)	CAAR (− 2, + 2)	SIZE and MV/BV matched BHAR		Propensity score matched BHAR	
			ABHAR 12	ABHAR 24	ABHAR 12	ABHAR 24
*Panel A*						
All sample	1.05%***	0.98%***	− 3.5%***	− 2.75%**	− 2.76%**	− 2.34%**
	(3.19)	(3.15)	(− 3.89)	(− 2.56)	(− 2.59)	(− 2.27)
*Panel B*						
Fraction of male directors > mean	0.22%***	0.28%***	− 3.21%***	− 3.37%***	− 2.68%**	− 2.45%***
	(2.15)	(2.19)	(− 3.75)	(− 3.86)	(− 2.53)	(− 3.62)
Fraction of male directors < mean	0.88%*	0.76%*	− 1.69%*	− 1.62%*	− 1.54%*	− 1.55%*)
	(1.39)	(1.32)	(− 1.54)	(− 1.51)	(− 1.47)	(− 2.47)
Mean difference *p* values	− 0.66%**	− 0.48%***	− 1.52%**	− 1.75%*	− 1.14%**	0.9%*
	(0.02)	(0.014)	(0.035)	(0.062)	(0.012)	(0.014)
*Panel C*						
Single	1.38%***	1.44%***	− 2.27%***	− 2.05%***	− 2.07%**	− 1.98%***
	(3.29)	(3.3)	(− 2.50)	(− 2.31)	(− 2.14)	(− 2.27)
Multiple	0.81%**	0.84%**	− 3.48%***	− 3.43%***	− 3.37%***	− 3.25%***
	(2.51)	(2.56)	(− 2.73)	(− 2.95)	(− 2.68)	(− 2.76)
Mean difference *p* values	0.6%**	0.60%***	1.21%**	1.38%*	1.3%***	1.27%***
	(0.041)	(0.007)	(0.02)	(0.09)	(− 0.017)	(0.013)
*Panel D*						
Cash	0.95%**	0.86%***	− 2.88%***	− 2.91%***	− 2.73%***	− 2.85%***
	(1.38)	(1.26)	(− 2.94)	(− 3.01)	(− 2.86)	(− 2.94)
Stock	1.78%**	1.96%**	− 1.53%**	− 1.61%**	− 1.59%**	− 1.57%**
	(0.82)	(0.89)	(− 1.75)	(− 1.81)	(− 1.71)	(− 1.73)
Mean difference *p* values	− 0.83%**	− 1.1%**	− 1.35%**	− 1.3%**	− 1.14%**	− 1.28%**
	(0.028)	(0.03)	(0.036)	(0.028)	(0.025)	(0.024)
*Panel E*						
Diversifying	1.27%***	1.31%***	− 3.1%***	− 3.17%***	− 2.95%***	− 2.46%***
	(3.17)	(3.28)	(− 3.09)	(− 2.76)	(− 3.19)	(− 2.88)
Related	1.48%**	1.56%**	− 1.6%**	− 1.96%***	− 1.67%**	− 1.73%***
	(3.08)	(3.12)	(− 2.56)	(− 3.02)	(− 2.62)	(− 2.97)
Mean difference *p* values	− 0.21%**	− 0.25%**	− 1.5%**	− 1.21%**	− 1.28%**	− 0.73%**
	(0.038)	(0.043)	(0.02)	(0.024)	(0.02)	(0.024)

Panel A of the reports the short run average CARs denoted as CAARs in the event windows (1, + 1) and (− 2, + 2) and long run post-merger average BHARs denoted as ABHARs in the 12 months and 24 months after merger completion for the whole sample. Panel B presents the subsample of CAARs and ABHARs after dividing the sample between the above mean and below mean of fraction of male directors. Panel C also shows the subsample of CAARs and ABHARs after dividing the sample between single and multiple acquirers. Panels D and E shows the subsample of CAARs and ABHARs after dividing the sample between cash and stock and diversifying and related deals respectively. ***p < 0.01, **p < 0.05, *p < 0.1

The post-merger ABHARs of the combined firms are negative. The ABHARs are significant at 1% levels for both the 12-months and 24-months post the merger completion date. The negative ABHARs are consistent with past M&A studies on post-merger performance that show negative shareholder wealth in the post-merger period (Agrawal et al. 1992; Megginson et al. 2004; Boateng et al. 2017; Cao et al. 2019).

The results of the CAARs and ABHARs for the subsample of firms where the fraction of male directors is greater than its mean and the subsample of firms where the fraction of male directors is less than its mean are shown in Panel B. The CAARs are positive and lower for the subsample where the fraction of male directors is greater than its mean, compared to the subsample where the fraction of male directors is less than its mean. This lends support to our hypothesis 1a that a higher fraction of male directors in the boardroom leads to poor short-term performance. The long-run post-merger results show that ABHARs are negative across both the subsamples for the fraction of male directors but the negative effect appears pronounced for firms with a higher fraction of male directors than the mean, thus lending support to our hypothesis 1b. The mean differences as shown in Panel B are statistically significant at the 1%, 5% and 10% levels. Taken together, this finding suggests that a higher fraction of male directors on the board tends to increase board overconfidence with detrimental implications for M&A outcomes. This result supports the findings of Huang and Kisgen (2013) that reported boards with male executives generate 2% lower announcement period returns.

#### Merger performance and overconfidence measured by merger frequencies

Panel C of Table 5 shows the CAARs and ABHARs for the subsample of firms categorised as single acquirers or multiple acquirers. As mentioned in Sect. 3, multiple acquirers are firms that conduct five or more acquisitions within a three-year period and the rest are single acquirers. The findings indicate that CAARs are positive across both the subsamples but greater for single acquirers across both the (− 1, + 1) and (− 2, + 2) event windows than the multiple acquirers, thereby supporting hypothesis 2a. All these acquirer CAARs are significant at either 1%, 5% or 10% level. The ABHARs are negative across both the subsamples but significantly worse for multiple acquirers than for single acquirers, again supporting hypothesis 2b. The ABHARs in Panel C are statistically significant at either 1% or 5% levels. These results are consistent with the studies by Doukas and Petmezas (2007) and Billet and Qian (2008). The results lend support to our hypotheses 2a and 2b that multiple acquisitions are due to managerial overconfidence and lead to poorer returns.

#### Merger performance and overconfidence measured by merger characteristics

Panel D of Table 5 shows that acquirer CAARs are lower for cash-financed M&A compared to stock-financed M&A (0.95% vs. 1.78%) for the event window (− 1, + 1) and (0.86%, vs. 1.96%) for the event window (− 2, + 2). The mean differences in CAARs between cash-financed and stock-financed M&As are negative and statistically significant. The results for ABHARs are similar to CAARs. As Panel D shows the ABHARs are higher for stock-financed deals than for cash-financed deals (− 1.53% vs. − 2.88%) for ABHAR 12 and (− 1.61% vs. − 2.91%) for ABHAR 24, although all the ABHARs are negative and statistically significant. These results support our hypotheses 3a and 3b that overconfident boards with ample internal resources use cash and earn lower announcement period abnormal returns and poor post-merger return as these M&As are driven by managerial hubris. These results are consistent with the findings by Brown and Sarma (2007), Malmendier and Tate (2008) and Ferris et al. (2013). However, our results are inconsistent with Loughran and Vijh (1997), which is based on US data and they have reported that cash mergers generate positive returns and stock mergers generate negative returns in the post-merger period. The difference in results could be explained by differences in the M&A markets in the US and the UK. As discussed before, in contrast to the US market, the vast majority of M&A deals in the UK are private firms. Given that investors may have less information about private targets, they will react negatively when companies spend cash on such M&A.

Panel E of Table 5 shows the short-term CAARs and 12-months and 24-months ABHARs in terms of related and diversifying M&A. The results show that acquirer CAARs for related M&A are higher than diversifying M&As (1.48% vs. 1.27%) for the event window (− 1, + 1) and (1.56% vs. 1.31%) for the event window (− 2, + 2). The results for long-term ABHARs are similar to CAARs. As Panel E shows, the ABHARs are higher for related deals than for diversifying deals (− 1.6% vs. − 3.1%) for ABHAR 12 and (− 1.96% vs. − 3.17%) for ABHAR 24 and all the ABHARs are negative and statistically significant. These results support our Hypotheses 4a and 4b that overconfident boards engage in diversifying M&As and that leads to lower shareholder wealth creation. These findings are consistent with Morck et al. (1990), Ferris et al. (2013) and Doukas and Petmezas (2007).

The announcement period results are shown in Table 6 (Panel A) for multiple acquirers with a first deal and multiple acquirers with five or more deals across two short-run announcement period event windows, i.e., CAAR (− 1, + 1) and CAAR (− 2, + 2). The results show that multiple acquirers’ first deal generates higher returns than multiple acquirers with five or more deals across both the event windows and these results are significant at either 1% or 5% level. These results lend support to Hypothesis 2a that multiple acquisitions are driven by managerial overconfidence, emanating from self-attribution bias, which led to lower announcement period returns for multiple acquirers with first deals compared to five or more deals. These results are consistent with the findings reported by Doukas and Petmezas (2007) and Billet and Qian (2008).

**Table 6:** Merger performance and overconfidence measured by merger characteristics

CAAR (− 1, + 1)	All	CAAR (− 2, + 2)	All
*Panel A*			
Multiple acquirer: 1st deal	1.21%**	Multiple acquirer: 1st deal	1.81%***
	(2.65)		(3.41)
No. of deals	84	No. of deals	80
Multiple acquirers: 5 or more deals	0.58%***	Multiple acquirers: 5 or more deals	0.53%***
	(3.72)		(3.67)
No. of deals	173	No. of deals	172
Mean difference *p* values	0.63%**	Mean difference *p* values	1.28%***
	(0.014)		(0.008)

12-month ABHAR	All	24-month ABHAR	All
*Panel B*			
Multiple acquirer: 1st deal	− 3.08%*	Multiple acquirer: 1st deal	− 3.12%***
	(− 1.36)		(− 2.26)
No. of deals	49	No. of deals	41
Multiple acquirers: 5 or more deals	− 3.45%***	Multiple acquirers: 5 or more deals	− 3.48%***
	(− 3.32)		(− 2.96)
No. of deals	271	No. of deals	271
Mean difference *p* values	0.37%	Mean difference *p* values	0.36%
	(0.04)		(0.02)

Panel A of the table shows the average CARs denoted as CAARs in the event windows (− 1, + 1) and (− 2, + 2) where the sample is divided between Multiple acquirers’ first deal and Multiple acquirers’ with 5th or more deals. Panel B shows the post-merger average BHARs denoted as ABHARs in the 12 months and 24 months post-merger period where the sample is divided between multiple acquirers’ first deal and multiple acquirers with 5 or more deals. We have presented the t-statistics are shown in parentheses. ***, ** and * are 1%, 5% and 10% significance levels respectively

Results for multiple acquirers with first deals and multiple acquirers with five or more deals for post-merger BHARs are shown in Table 6 (Panel B). The BHARs in both cases are negative and significant at 1% level. This again supports the findings by Doukas and Petmezas (2007). These findings are consistent with Hypothesis 2b which states that multiple acquirers engage in subsequent M&A out of managerial overconfidence due to self-attribution bias and this leads to significant wealth losses.

### Multiple regression results

Table 7 presents the results of the multiple regressions with short-run CARs (− 1, + 1) and (− 2, + 2) and 12-months and 24-months post-merger BHARs as dependent variables. The findings indicate that the coefficients of the independent variable, fraction of male directors, are negative and statistically significant at 1% level for both short-run event windows (− 1, + 1) and (− 2, + 2) and long-run 12-months and 24-months post-merger BHARs. These results confirm the findings of univariate analysis reported in Table 5 and provide support for the Hypotheses 1a and 1b that a greater fraction of male directors (i.e., overconfidence) generates lower returns in the short as well as in the long run. The coefficients of the multiple acquisitions (i.e., proxy for overconfidence) are negative and significant at 1% and 5% levels for short and long runs respectively, suggesting that multiple acquisitions destroy firm value which is consistent with the managerial overconfidence hypothesis.

**Table 7:** Multivariate analysis results for overconfidence and merger performance

	Panel A	Panel B	Panel C	Panel D	Panel E	Panel F	Panel G	Panel H
	CAR (− 1, + 1)	CAR (− 1, + 1)	CAR (− 2, + 2)	CAR (− 2, + 2)	BHAR 12	BHAR 12	BHAR 24	BHAR 24
*Independent variables*								
Fraction of male directors	− 1.85***	− 2.22***	− 1.45***	− 1.52***	− 1.77***	− 1.69**	− 2.83***	− 2.78***
	(− 3.16)	(− 3.34)	(− 3.23)	(− 3.31)	(− 3.42)	(− 2.75)	(− 3.61)	(− 3.55)
Multiple	− 1.38***	− 1.35***	− 1.29***	− 1.31***	− 1.37**	− 1.51**	− 1.45**	− 1.56**
	(− 3.29)	(− 3.24)	(− 3.15)	(− 3.18)	(− 2.57)	(− 2.64)	(− 2.93)	(− 2.99)
Fraction of male directors*Multiple		− 1.28**		− 1.23**		− 1.26**		− 1.37**
		(− 2.69)		(− 2.48)		(− 2.53)		(− 2.81)
Cash	− 1.36	− 1.47	− 1.28	− 1.42	− 0.89	− 0.94	− 0.91	− 0.97
	(− 0.64)	(− 0.78)	(− 0.59)	(− 0.72)	(− 0.75)	(− 0.81)	(− 0.78)	(− 0.86)
Stock	0.17	0.12	0.21	0.18	0.15*	0.22*	0.12*	0.17*
	(0.92)	(0.79)	(1.02)	(0.85)	(1.78)	(1.85)	(1.73)	(1.77)
Related	0.89**	0.93**	0.92**	0.97**	0.21***	0.28***	0.17***	0.19***
	(2.51)	(2.52)	(2.56)	(2.64)	(3.52)	(3.56)	(3.42)	(3.47)
*Control variables*								
CEO duality	− 1.19	− 1.22	− 1.32	− 1.37	− 1.93**	− 1.95**	− 2.11**	− 2.18**
	(− 0.96)	(0.98)	(− 1.11)	(− 1.18)	(− 2.22)	(− 2.28)	(− 2.43)	(− 2.48)
Board size	0.021	0.027	0.015	0.019	0.012	0.017	0.026	0.021
	(0.68)	(0.75)	(0.59)	(0.64)	(0.38)	(0.43)	(0.49)	(0.37)
Board Independence	0.45**	0.52**	0.47**	0.52**	0.49**	0.56**	0.41**	0.38**
	(2.46)	(2.56)	(2.49)	(2.55)	(2.52)	(2.59)	(3.02)	(2.95)
Tobin's Q	0.58	0.52	0.48	0.51	0.93***	0.89***	0.93***	0.98***
	(0.87)	(0.81)	(0.79)	(0.82)	(3.45)	(3.38)	(3.41)	(3.47)
Leverage	− 1.92	− 1.91	− 1.91	− 1.87	− 2.16**	2.09**	− 2.16**	− 2.24**
	(− 1.11	(− 1.12)	(− 1.16)	(− 1.13)	(− 2.22)	(− 2.17)	(− 2.34)	(− 2.39)
Constant	1.41***	1.45***	1.41***	1.52***	− 0.48***	− 0.42***	− 0.73***	− 0.76***
	(3.57)	(3.62)	(3.61)	(3.68)	(3.37)	(3.33)	(3.52)	(3.57)
R squared	0.65	0.61	0.57	0.61	0.64	0.56	0.68	0.65
No. of observations	725	725	725	725	693	693	690	690

The results show the determinants of short run announcement period returns and long run post-merger returns where the dependent variables are short run CARs for the windows (− 1, + 1) and (− 2, + 2) and 12 months and 24 months post-merger BHARs. The figures in parenthesis are the t-statistics. ***, ** and * represent 1%, 5% and 10% significance levels respectively

We carried out further analysis by incorporating the interaction term between the directors and multiple acquisitions (fraction of male directors*multiple acquisitions). The coefficients for this interaction term are negative and significant at the 5% level, as shown across all the panels, thus supporting our earlier findings.

Cash-financed acquirers generated wealth losses, as evidenced by the negative coefficients in the short run and long run. However, these are not statistically significant. The coefficients of related deals are positive and significant at 5% and 1% levels respectively for short-run CAARs and long-run BHARs, confirming Hypotheses 4a and 4b which state that related deals are less value destructive in comparison to diversified deals.

### Robustness tests

To test the robustness of long term BHARs we have conducted further regression using propensity score matched BHARs. The results are shown in Table 8. The regression results for the 12-months and 24-months propensity score matched BHARs as dependent variables are consistent with the main regression results reported in Table 7.

**Table 8:** Robustness check

	Panel A	Panel B
	BHAR 12	BHAR 24
*Independent variables*		
Fraction of male directors	− 2.67**	− 2.59**
	(− 2.95)	(− 2.73)
Multiple	− 1.42**	− 1.51**
	(− 2.89)	(− 2.93)
Fraction of male directors*Multiple		− 1.35**
		(− 2.75)
Cash	− 0.84	− 0.93
	(− 0.72)	(− 0.81)
Stock	0.16*	0.22*
	(1.78)	(1.82)
Related	0.24***	0.27***
	(3.58)	(3.61)
*Control variables*		
CEO duality	− 2.16**	− 2.25**
	(− 2.46)	(− 2.51)
Board size	0.021	0.017
	(0.43)	(0.35)
Board independence	0.48**	0.51**
	(3.10)	(3.15)
Tobin's Q	0.98***	0.92***
	(3.47)	(3.39)
Leverage	− 2.26**	− 2.29**
	(− 2.47)	(− 2.52)
Constant	− 0.66**	− 0.83**
	(2.73)	(2.91)
R squared	0.72	0.76
No. of observations	684	684

The results show the determinants of short run announcement period returns and long run post-merger returns where the dependent variables 12-months and 24-months propensity score matched BHARs. The figures in parenthesis are the t-statistics. ***, ** and * represent 1%, 5% and 10% significance levels respectively

## Conclusion

This study provides new evidence on the implications of managerial overconfidence for M&A performance. Using three measures of overconfidence, namely, fraction of male directors, multiple acquisitions and merger characteristics, the results of the study show that managerial overconfidence has a significant harmful impact on M&A with regard to shareholders’ wealth creation.

In relation to our first measure of overconfidence, i.e., the fraction of male directors, the results indicate that short-run CAARs are lower for acquirer firms with a higher fraction of male directors. Similarly, results of the study also show that firms with a higher fraction of male directors demonstrate poor post-merger performance. This finding extends the existing literature by providing a new measure of overconfidence and implies that boards dominated by male directors tend to exhibit overconfidence. Moreover, these overconfident boards are involved in value destroying M&A. We argue that policy makers should take the number of male directors into account in reforming corporate governance codes in the context of M&A.

With regard to our second measure of managerial overconfidence, which is multiple acquisitions, results of this study indicate that multiple acquisitions generate lower CAARs than single acquisitions. It is argued that this can be attributed to self-attribution bias, i.e., managers tend to be overconfident after succeeding in the first deals and hence engage in multiple acquisitions that lead to poor performance in subsequent periods.

Finally, this study finds that overconfident managers with abundant internal resources use cash as the method of payment. Such cash-financed M&As earn lower CAARs in comparison to stock-financed M&As and such deals also demonstrate greater post-merger wealth losses. Therefore, we argue that the method of payment in an overconfident board could be an indicator of M&A performance. With this in mind, policy makers and shareholders of acquiring firms should give due consideration to how M&A is paid for in the case of overconfident boards as it has important implications for post-merger shareholders’ wealth. In addition to this, results of this study also indicate that overconfident managers conduct diversifying acquisitions where they rely more on their own judgement of potential synergies from M&A. However, such a behaviour leads to higher wealth losses in the long run.

## Data Availability

The data that support the findings of this study are available from the corresponding author, upon reasonable request. The data on M&A and merger characteristics have been obtained from the SDC database. All other data on share price and firm characteristics have been collected from Datastream.

## Footnotes

1 In this study, the terms, acquisitions, mergers and takeovers have been used interchangeably.

2 See Bradley et al. (1988), Moeller et al. (2004) and Antoniou et al. (2007).

3 See Rau and Vermaelen (1998), Megginson et al. (2004) and Datta et al. (2013).

4 Agrawal, Jaffe, and Mandelker (1992), Loughran and Vijh (1997) and Mitchell and Stafford (2000) are based on US data whereas our study is based on the UK data.

5 This belief is also in line with Heaton (2002), who asserts that managers conduct numerous projects due to their overconfidence.

6 Mergers and acquisitions involving UK companies, annual overview—Office for National Statistics.

7 https://www.ey.com/en_uk/ccb/united-kingdom-mergers-acquisitions.

8 This result supports the findings of Billet and Qian (2008) on US M&A.

9 Doukas and Petmesaz (2007) measure overconfident managers as those managers who engage in frequent acquisitions, that is five or more acquisitions within a span of five years.

10 This supports the findings of Hansen (1987), who showed that the choice of cash payment indicates that acquirers presume to be more knowledgeable about gauging a target’s value.
